# Characterization of Fetal Thyroid Levels at Delivery among Appalachian Infants

**DOI:** 10.3390/jcm9093056

**Published:** 2020-09-22

**Authors:** Madison N. Crank, Jesse N. Cottrell, Brenda L. Mitchell, Monica A. Valentovic

**Affiliations:** 1 Department of Obstetrics and Gynecology, Joan C. Edwards School of Medicine, Marshall University, Huntington, WV 25755-9310, USA; crank26@live.marshall.edu (M.N.C.); cottrellje@marshall.edu (J.N.C.); Dawleyb@marshall.edu (B.L.M.); 2 Department of Biomedical Sciences Pharmacology/Toxicology Research Cluster, Joan C. Edwards School of Medicine, Marshall University, Huntington, WV 25755-9310, USA

**Keywords:** thyroid function tests, Appalachia, fetus, pregnancy

## Abstract

Thyroid disorders are a frequently encountered issue during pregnancy and a cause of maternal and fetal morbidity. In regions like Appalachia that are particularly susceptible to health disparities, descriptive studies are needed to assist in identifying pathologic derangements. We sought to characterize fetal thyroid hormone levels at delivery and investigate whether or not maternal demographic characteristics affect the prevalence of neonatal thyroid disease. A cross-sectional analysis was conducted on 130 pregnant women recruited from the Tri-State region, incorporating areas of Kentucky, Ohio, and West Virginia. Total triiodothyronine (T3) (*p* = 0.4799), free T3 (*p* = 0.6323), T3 uptake (*p* = 0.0926), total thyroxine (T4) (*p* = 0.8316), free T4 (*p* = 0.0566), and Thyroid stimulating hormone (TSH) (*p* = 0.8745) levels were comparable between urban and rural newborns. We found no effect of hypertension status or nicotine levels on fetal umbilical cord thyroid hormone levels. Maternal diabetic status was associated with lower T4 (*p* = 0.0099) and free T4 (*p* = 0.0025) levels. Cotinine affected levels of T4 (*p* = 0.0339). In regard to maternal Body Mass Index (BMI), there was an increase in total T3 as BMI increased (*p* = 0.0367) and no significant difference in free T3, T3 uptake, T4, free T4, or TSH. There was a negative correlation between TSH and 1 min Apgar scores (*p* = 0.0058). Lead and cadmium have been implicated to alter TSH levels, but no correlation was found in our study (r^2^ = 0.0277). There were no differences in cord blood between urban (37.3 ± 10.3 fmol/ug DNA) and rural (70.5 ± 26.8 fmol/ug DNA) benzo(a)pyrene DNA adducts (*p* = 0.174). Thyroid disorders present a unique opportunity for the prevention of perinatal morbidity and mortality, since maternal treatment, as well as maternal demographic characteristics, can have direct fetal effects.

## 1. Introduction

Thyroid disorders are common during pregnancy and are the second most common endocrine condition in women of childbearing age, following diabetes [1,2]. Previous studies have analyzed the effect of diabetes, hypertension, obesity, and urinary nicotine and cotinine levels on thyroid function in various populations without taking into account the participants’ rural or urban backgrounds.

Recent data suggest that the Appalachian region, which includes all of West Virginia, is particularly susceptible to health disparities in terms of access to healthcare and mortality rates as a result of common chronic medical issues and living conditions. These disparities exist between those living in predominantly urban compared to rural environments, as well as the Appalachian region as a whole [3,4,5,6,7]. To our knowledge, no one has studied how neonatal thyroid hormone levels differ between mothers from rural and urban areas to characterize the levels and to investigate whether or not a disparity exists. Thyroid disorders present a unique opportunity for the prevention of perinatal morbidity and mortality, as maternal treatment can have direct fetal effects. While newborn screening for thyroid hormone levels is important for maintaining proper development postnatally, the thyroid gland also plays a critical role in fetal development in utero.

To date, there have been no studies examining fetal thyroid hormone levels from umbilical cord blood at the time of delivery in Appalachian infants. Pediatric thyroid hormone reference ranges are available online for infants greater than 1 day old, but the data are scarce [8]. The purpose of our study was to perform a cross-sectional analysis in an attempt to better understand the role of maternal demographic characteristics on fetal thyroid hormone levels. We sought to characterize fetal thyroid hormone levels at delivery among Appalachian infants and investigate whether or not there is a difference in the prevalence of altered hormone status in rural Appalachia when compared to that of the urban regions of the tri-state area. This study further evaluated whether cigarette smoking, diabetes, or obesity can influence thyroid hormone levels. A component of cigarette smoke is benzo(a)pyrene (BaP), a polycyclic aromatic hydrocarbon that is activated by cytochrome P450 to form reactive epoxides that can covalently bind to DNA [9]. This study also examined whether DNA adducts were detectable in umbilical cord blood.

## 2. Materials and Methods

### 2.1. Patients

**Figure 1:**
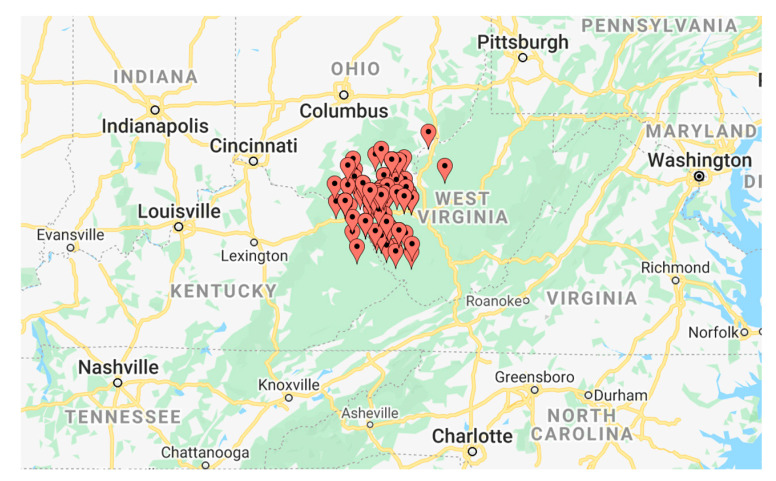
Map of subjects depicted on U.S. map of Eastern United States.

**Figure 2:**
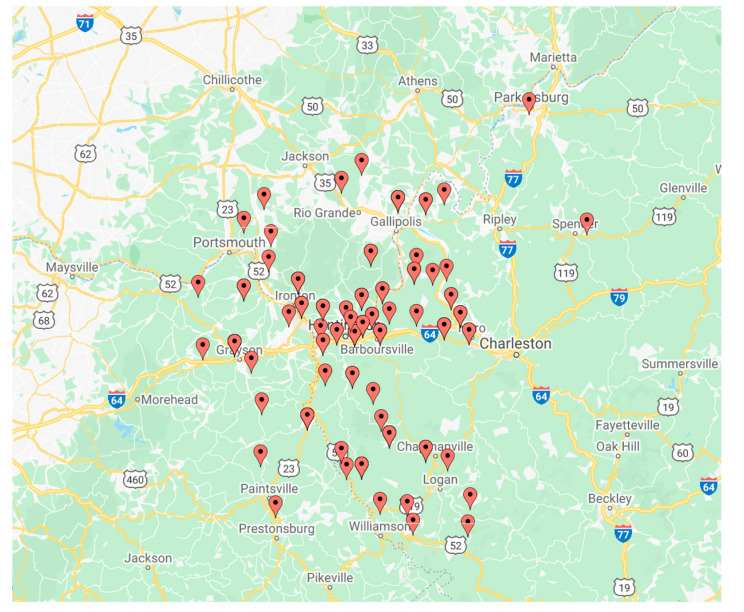
Depiction of study subjects based on Rural-Urban Commuting Codes RUCA zip codes. All individuals located in West Virginia, Ohio, or Kentucky.

A cross-sectional analysis was conducted in 130 pregnant women. Consenting participants were recruited upon presentation to the Cabell Huntington Hospital Labor and Delivery unit in a consecutive manner. The geographic region of the subjects was the Tri-State region, which incorporates areas of Kentucky, Ohio, and West Virginia (Figure 1 and Figure 2). The collection of samples took place between 15 June 2013 and 1 February 2014. Demographic information was collected from the patients during admission to Labor and Delivery at Cabell Huntington Hospital. Lab values were extracted from each patient’s electronic medical record. Rural and urban locations were based on Rural-Urban Commuting Area (RUCA) Codes, determined by the U.S. census tracts using measures of population density, urbanization, and daily commuting, which were then applied to U.S. postal service zip codes (Table 1).

### 2.2. Sample Collection

Inclusion criteria were that participants must have had a successful vaginal delivery or cesarean section and sufficient cord blood must have been obtained. Patients were excluded for non-collection of cord blood or inadequate collection. Any patient with a history of thyroid disorder or taking levothyroxine was also excluded. Prior to the start of the project, the study protocol was approved by the Human Research and Ethics Institutional Review Board (IRB) of Marshall University. The analysis included levels of thyroid stimulating hormone (TSH) levels, total and free thyroxine (T4), total and free triiodothyronine (T3), and T3 uptake. The fetal umbilical cord blood thyroid hormone levels were analyzed in the Cabell Huntington Hospital laboratory by Siemens Centaur (T3, T3 uptake, and free T3 levels) and Siemens Vista (T4, free T4, and TSH levels), trademarks of Siemens Healthcare Diagnostics Incorporated, located in Tarrytown, NY, USA. The samples were also analyzed for nicotine and cotinine.

The umbilical cord blood was also analyzed for cadmium and lead by The Great Plains Laboratory, Inc. located in Lenexa, KS, USA. Higher cadmium levels have been detected in tobacco smokers, and lead is associated with primary tobacco use as well as second-hand smoke [10,11]. Both metals have been implicated to alter TSH levels and cause thyroid dysfunction, but more studies are needed to elucidate the underlying pathologic mechanisms [12,13]. According to the lab protocol, The Great Plains Laboratory analyzes the elements in whole blood by inductively coupled plasma mass spectroscopy following specimen digestion with nitric acid in a closed containing microwave oven system. The procedure measures the total concentration of an element in whole blood, regardless of the biochemical form and regardless of partitioning of the element in blood fractions.

### 2.3. DNA Adduct Analysis

Cord blood (15 mL) was collected and processed with PAX gene Blood DNA Kits to isolate DNA. DNA was quantitated by a nanodrop. The Benzo(a)pyrene (BaP) tetrols were hydrolyzed from DNA by heating for 4 h at 90 ℃. The BaP tetrols were analyzed by HPLC based on methods of Alexandrov and associates [14] with a Beckman and Waters High Pressure Liquid Chromatography (HPLC) system with a Lichrosorb 5 micron C18 column (250 mm column length and 4.0 mm inner diameter) with a flow rate of 0.5 or 1 mL/min for the Waters and Beckman systems, resp. The mobile phase was isocratic 55% aqueous methanol. Tetrols were identified by a Shimadzu fluorescent detector with an excitation of 344 nm and an emission of 398 nm. A standard curve of Benzo(a)pyrene-r-7, t-8, t-9, t-10-tetrahydrotetrol(+/−) I-2 in methanol was used for the calculation of tetrol levels.

### 2.4. Statistical Analysis

Comparisons and *p*-values were by one-way ANOVA using JMP from SAS located in Cary, NC, version 15.0. Differences between groups were analyzed using a 95% confidence interval and considered statistically different at *p* < 0.05.

## 3. Results

Rural and urban study group demographics were not statistically different, and values were combined for maternal age, insurance status, race, length of hospital stay, employment status, marital status, prenatal vitamin use, alcohol or substance use, presence of hypertension or diabetes, mean gestational age, estimated blood loss, need for labor induction or cesarean section, mean hemoglobin or hematocrit, mean white blood cell (WBC) count, or mean platelet count (Table 2). There were no differences in infants born to rural versus urban subjects regarding sex, mean 1 min and 5 min Apgar scores, mean birth weight, mean head circumference, mean length at birth, mean length of hospital stay, or percentage needing transferred to the neonatal intensive care unit (NICU). The combined data from urban and rural groups are provided (Table 3 and Table 4).

Mean umbilical cord blood thyroid hormone levels are also listed (Table 5). Total T3, free T3, T3 uptake, total T4, free T4, and TSH umbilical cord blood levels were comparable between urban and rural newborns.

We found no significant effect of hypertension status on fetal umbilical cord thyroid hormone levels (Table 6). Although only 10 patients had diabetes, maternal diabetic status was associated with significantly lower thyroid hormone levels T4 (*p* = 0.0099) and free T4 (*p* = 0.0025) (Table 7). There was no significant association of nicotine with thyroid hormone levels, including total T3 (*p* = 0.3893, 1.00 nmol/L), free T3 (*p* = 0.1825, 0.78 ng/dL), T3 uptake (*p* = 0.3712, 0.04%), T4 (*p* = 0.3423, 0.09 mcg/dL), free T4 (*p* = 0.5004, 0.74 ng/dL), or TSH (*p* = 0.9723, −0.00 mU/L) (Table 8). Cotinine did have an interaction with free T4 levels (*p* = 0.0339, 49.58 mcg/dL) but had no significant effect on total T3 (*p* = 0.1413, 36.48 nmol/L), free T3 (*p* = 0.0238, 28.06 ng/dL), T3 uptake (*p* = 0.7227, −0.36%), T4 (*p* = 0.0822, 3.33 ng/dL), T4 (*p* = 0.0822, 3.33 mcg/dL), or TSH (*p* = 0.2358, −0.85 mU/L) (Table 9).

In regard to maternal Body Mass Index (BM)I, our study showed an increase in total T3 as BMI increased (*p* = 0.0367) and no difference in free T3, T3 uptake, T4, free T4, or TSH.

There was a negative correlation between fetal TSH and 1 min Apgar scores (*p* = 0.0058). All other measured fetal outcomes (height, head circumference, birth weight, 5 min Apgar score, baby length of hospital stay) did not show any statistically significant correlation with thyroid hormones at the time of delivery.

Lead and cadmium have been implicated with tobacco smoking and have been shown to alter TSH levels, but no correlation was found in our study (r^2^ = 0.0277) [12,13]. The lack of a correlation of metals may be attributed to mothers who were asked if they use tobacco but were not asked to identify the time lapse since the last use of a tobacco product. HPLC analysis for BaP-DNA adducts is a biomarker for sufficient exposure to BaP to result in DNA covalent binding. A total of 21 blood samples had detectable levels of BaP DNA adducts. There were no differences in BaP levels between urban (37.3 ± 10.3 fmol/ug DNA) and rural (70.5 ± 26.8 fmol/ug DNA) for DNA adducts (*p* = 0.174).

## 4. Discussion

The Appalachian Region includes all of West Virginia and parts of 12 other surrounding states, covering 205,000 square miles and containing over 25 million people. Forty-two percent of the Appalachian Region population is rural, while only 20% of the nation’s population is rural. Across the region, poverty and unemployment rates, incomes, and education levels continue to fall behind the rest of the nation’s performance in these areas, despite efforts to bridge the gap. A few key indicators from a recent report on health disparities in Appalachia include higher mortality rates as a result of heart disease, cancer, stroke, diabetes, drug overdose, and suicide when compared to the rest of the nation, as well as a higher prevalence of depression, fewer healthcare professionals, and worse living conditions in the region [15]. Despite recent improvements in several of these categories over the past two decades, our progress is insufficient when compared to that of the rest of the nation. We want to further investigate rural health disparities and how they affect neonatal health and thyroid function. A gap in the knowledge exists regarding whether or not babies born in the Appalachian region have an abnormal thyroid status, when comparing babies born to mothers from rural versus urban areas.

The thyroid gland plays a critical role in fetal development [16,17]. The developing fetus is reliant on transplacental passage of thyroid hormone until the 12th week of gestation, at which point thyroid hormones T3 and T4 begin to be produced by follicular cells of the thyroid gland. Clinically significant levels of T4 are produced by 18–20 weeks of gestation, while T3 significantly increases at 30 weeks of gestation. Postnatally, TSH, T4, and T3 levels rise abruptly, rapidly dwindle for the first five days of life, then gradually fall until about 1 month of life in both term and preterm infants. However, in preterm babies, the change in hormone levels is proportionally smaller [18].

T3 is the metabolically active form of thyroid hormone and occupies nuclear receptors to mediate the actions of thyroid hormone, enabling proper brain, liver, and skeletal development [19]. Without thyroid hormone, newborns may present with skeletal abnormalities due to a lack of endochondral and intramembranous ossification, longitudinal bone growth, and skeletal maturation. Thyroid hormones also regulate the expression of many genes important for embryonic central nervous system development, and newborns can develop permanent intellectual disability in the absence of timely and proper thyroid hormone supplementation [19,20,21,22].

Universal screening for congenital hypothyroidism obtained by a heel stick has helped to eliminate the devastating consequences of this condition, which affects 1 in 2000 to 1 in 4000 newborns [23]. Additional screening tests are recommended for preterm infants to avoid false negative results from a delayed rise in serum TSH in these newborns [24]. Reference intervals for thyroid hormone levels in infants between 1 day and 1 month old include 1.75–5 mIU/L TSH, 1.05–1.9 mg/dL T4, and 3.5–4.75 pg/mL T3 in females, and 3.7–4.5 pg/mL T3 in males. No reference intervals are available for infants less than 1 day old [8]. Our neonate’s thyroid hormone levels were not comparable to these reference ranges, as mean TSH levels (8.64 mU/L) were higher and mean free T4 (0.97 ng/dL) and free T3 (1.44 ng/dL) levels were lower. The variability in results may be due to the recognized changes occurring at birth [18]. However, TSH levels were comparable to a study by Shields and associates that reported TSH levels of 8.02 mIU/L in a cohort of their study examining blood levels during pregnancy and in umbilical cord blood [25].

Attempting to account for thyroid disorder in relation to socioeconomic class, Talat, et al. did not show any association between income level and thyroid hormone levels [26]. However, they did note that most of the subjects in their study had free access to healthcare and regular checkups, which may have affected the results. In another study, a Pakistani population’s prevalence of overt hypothyroidism was approximately 4%, while a study on northern Indian women showed a prevalence of 7%. The authors attributed differences in diet to partially account for this apparent disparity [26,27]. Furthermore, studies in China have shown variation in thyroid hormone levels in different races [28].

A recent cross-sectional study by Velasco et al. examined the relationship between maternal and neonatal thyroid parameters, the effects of those hormone levels on birth outcomes, and the specific role of thyroid hormone in the regulation of fetal growth in low-risk pregnancies lacking any condition of obstetric or perinatal risk versus high-risk pregnancies. They found that maternal thyroid hormones may have a significant impact on fetal growth regulation. Thyroid function and iodine status at birth was affected by fetal iodine status, placental function, amniotic fluid levels, labor onset, and mode of delivery. Mean urinary iodine concentration was lower in high-risk pregnancies, and non-consumption of iodine supplements was associated with preterm births. However, iodine concentration in amniotic fluid was higher in high-risk pregnancies and had a negative correlation with birth weight. A negative association between maternal free T4 and neonatal birth weight could be explained by the hypothesized effect of compromised transplacental transfer of thyroid hormone: The more impeded the transfer of thyroid hormone is, the higher the maternal free T4 will be and the lower the neonatal thyroid hormone level will be. All thyroid parameters were higher in the low-risk pregnancy group besides free T4, which was similar between the low- and high-risk groups, with a positive correlation between maternal and neonatal thyroid hormone levels across the board. They concluded that an unfavorable environment in utero can limit the availability of free T4 in cord blood and limit fetal iodine metabolism, especially in small fetuses [29]. Due to the limited number of neonatal thyroid studies available, our study examined whether or not specific maternal demographics impacted neonatal thyroid hormone levels. Our findings indicated that conditions of obesity and diabetes, which are more prevalent in the Appalachian region, were associated with an altered thyroid hormone status.

Previous studies have shown that maternal BMI may have an effect on thyroid function. Upon analysis of thyroid glands from electively terminated fetuses in the second trimester, Filis et al. found significant changes in thyroid function, increased fetal thyroid mass and plasma TSH levels, and changes in the expression of genes critical for proper thyroid development based upon maternal BMI [30]. Another study conducted on Zhuang ethnic pregnant women did not find any statistically significant correlation between BMI and overt hypothyroidism, but a BMI > 25 was associated with a higher risk of overt hypothyroxinemia, defined as having a normal TSH with free T4 levels in the lower 2.5th percentile, based on the reference range for each trimester [31]. However, our study did not show any association between BMI and thyroid disease.

In regard to fetal thyroid function and maternal diabetes, our study showed a significant difference with lower total T4 and free T4 in those with diabetes compared to those without diabetes (Table 7). In 2000, the prevalence of thyroid disease in the general population and non-diabetics was 6.6% and 10.8%, respectively, with hypothyroidism being the most common type of thyroid dysfunction [32]. Due to their increased risk of thyroid disorders, diabetics are screened regularly for thyroid abnormalities [32,33]. Additionally, a study in 2012 by Karakosta et al. showed a 4-fold increased risk of gestational diabetes associated with mothers who had a high TSH and thyroid antibodies in the first trimester of pregnancy [34]. Recent studies have revealed a potential link between thyroid dysfunction and the development of type 2 diabetes mellitus due to dysregulated intestinal glucose absorption, alterations in gene expression, and aberrant hepatic processing of glucose that can occur in the setting of thyroid disease. In fact, hypothyroidism and hyperthyroidism have been associated with the development of insulin resistance [35,36]. We were limited by our number of diabetic patients included in our study, and further study is needed in this area. However, the lower total T4 and free T4 in the umbilical cord blood of mothers with diabetes suggests that newborns should be evaluated for thyroid status during well baby and pediatric examinations.

Smoking during pregnancy may also have an effect on thyroid function. Based upon urinary cotinine levels, cigarette smoking has been associated with decreased TSH levels and increased TPO titers in a dose-dependent manner [37]. Proposed mechanisms to explain this finding are that smoke may interfere with the process of iodide transport and organification, or smoking may have a stimulatory effect on the thyroid gland, leading to higher levels of thyroxin-binding globulin and T3 concentrations and lower TSH concentrations due to negative feedback [37]. Similarly, a 2007 Swedish study demonstrated that smoke exposure, assessed by urinary cotinine levels and questionnaires, was associated with higher free T3 and total T3 and lower concentrations of TSH during pregnancy [38]. In our study, only cotinine levels affected T4. Cigarette smoking is associated with exposure to cadmium and lead, but in the present study, no differences were measured in lead or cadmium levels between smokers. The lack of a difference in metal levels in smokers may be due to the elapsed time between smoking and collection of umbilical cord blood. However, smoking was sufficient in some individuals to induce DNA adducts with BaP. This covalent binding reflects long-term exposure.

This is the largest study to date attempting to characterize the thyroid hormone levels at delivery of infants born in Appalachia. A strength of our study was the fairly homogenous patient population surveyed, robust numbers, and demographic information that was collected at the time of admission to labor and delivery. While our study was prospective in nature, due to the paucity of knowledge regarding the appropriate level of thyroid hormones at birth, more study is needed in this area. A follow-up of the infants whose cord blood was collected for this study is planned.

## 5. Conclusions

Thyroid disorders are a frequently encountered issue during pregnancy and a cause of both maternal and fetal morbidity. In regions like Appalachia that are particularly susceptible to health disparities, descriptive studies are needed to assist in identifying pathologic derangements. While screening of congenital hypothyroidism via TSH is standard for all infants, there is little data regarding total T4, free T4, free T3, and T3 uptake. Our study provides such data to help characterize fetal thyroid levels at delivery among Appalachian infants. Further studies are needed to determine how levels of thyroid hormones at delivery affect long-term health outcomes for infants born in Appalachia.

## Figures and Tables

**Table 1:** Rural-Urban Commuting Area Codes (RUCA) Distribution for the States of WV, KY, and OH.

RUCA Level	Count
1	51
2	11
2.1	3
2.3	1
4	5
5	10
5.2	1
6	2
7	2
7.3	15
8	4
8.3	1
9	1
9.1	1
10	11
10.4	2
10.5	4
10.6	5

**Table 2:** Maternal Information. Reported mean ± standard deviation (SD) for continuous variables or *n* (%) for categorical variables. 95% confidence interval.

	Mean with Standard Deviation
Age (years)	25.66 ± 5.14
BMI (kg/m^2^) *	31.7 ± 6.81
Insurance Type	
Private	52 (41%)
Public	72 (56%)
None	4 (3%)
Race:	
African-American	3 (2%)
Asian	1 (1%)
Not Specified	1 (1%)
White	125 (96%)
Length of Hospital Stay (days)	3.48 ± 2.34
Employment	
Employed	50 (38%)
Not Employed	80 (62%)
Marital Status	
Divorced	7 (5%)
Married	63 (48%)
Single	59 (45%)
Widow	1 (1%)
Alcohol Use	
Yes	1 (1%)
Denies	129 (99%)
Substance Abuse	
Yes	13 (10%)
Denies	117 (90%)
Hypertension	
No	100 (77%)
Yes	30 (23%)
Diabetes	
No	120 (92%)
Yes	10 (8%)
Prenatal Vitamin Use	
No	32 (25%)
Yes	98 (75%)

* BMI, Body Mass Index.

**Table 3:** Delivery Information. Reported mean ± SD for continuous variables or *n* (%) for categorical variables. 95% confidence interval.

	Mean with Standard Deviation
Gestational Age at Delivery (weeks)	38.55 ± 1.53
Estimate Blood Loss at the time of Delivery (mL)	410.92 ± 189.93
Induction/Non-Induction	
Induction	78 (60%)
Non-Induction	52 (40%)
Mode of Delivery	
Cesarean section	40 (31%)
Vaginal Delivery	90 (69%)
Hemoglobin (g/dL)	11.83 ± 1.22
Hematocrit (vol%)	35.53 ± 3.85
WBC (10^3^/mm^3^) *	11.51 ± 3.22
Platelets (10^3^/mm^3^)	202.28 ± 58.01

* White Blood Cell (WC).

**Table 4:** Fetal Information. Reported mean ± standard deviation (SD) for continuous variables or *n* (%) for categorical variables. 95% confidence interval.

	Mean with Standard Deviation or Number of Subjects in Subset
Gender	
Female	61 (47%)
Male	69 (53%)
Apgar 1 Minute	8.34 ± 0.91
Apgar 5 Minute	8.92 ± 0.32
Birth Weight (grams)	3260 ± 0.56
Head Circumference (cm)	34.28 ± 2.18
Length (cm)	50.23 ± 2.84
Length of Hospital Stay (days)	5.73 ± 8.23
NICU/WBN *	
NICU	24 (18%)
WBN	106 (82%)

* Neonatal Intensive Care Unit (NICU), Well Baby Nursery(WBN).

**Table 5:** Umbilical cord blood thyroid hormone levels. Reported mean ± SD. 95% confidence interval.

Parameters	Mean ± SD	Std. Error	Upper 95% Mean	Lower 95% Mean
Total T3 * (nmol/L)	0.51 ± 0.17	0.02	0.54	0.48
Free T3 * (ng/dL)	1.44 ± 0.35	0.03	1.50	1.38
T3 Uptake (%)	28.97 ± 4.17	0.37	29.69	28.25
T4 * (mcg/dL)	11.74 ± 2.25	0.20	12.13	11.35
Free T4 (ng/dL)	0.97 ± 0.18	0.02	1.00	0.94
TSH (mU/L)	8.64 ± 6.02	0.53	9.68	7.59

* *n* = 129.T3, Triiodothyronine; T4, thyroxine; TSH, Thyroid Stimulating Hormone

**Table 6:** Effect of hypertension on fetal thyroid hormones.

	No Hypertension (*n* = 100)		Hypertension (*n* = 30)		
	Mean	Std. Error	Mean	Std. Error	ANOVA F-Test *p*-Value
Total T3 (nmol/L)	0.52 *	0.02	0.47	0.03	0.1873
Free T3 (ng/dL)	1.45 *	0.03	1.40	0.63	0.5326
T3 Uptake (%)	28.65	0.41	30.03	0.76	0.1114
T4 (mcg/dL)	11.97 *	0.22	10.98	0.40	0.0341
Free T4 (ng/dL)	0.98	0.02	0.94	0.03	0.2806
TSH (mU/L)	8.73	0.60	8.31	1.10	0.7363

* *n* = 99. T3, Triiodothyronine; T4, thyroxine; TSH, Thyroid Stimulating Hormone

**Table 7:** Effect of diabetes on fetal thyroid hormones.

	No Diabetes (*n* = 120)		Diabetes (*n* = 10)		
	Mean	Std Error	Mean	Std Error	ANOVA F-Test *p*-Value
Total T3 (nmol/L)	0.50 *	0.02	0.54	0.06	0.5702
Free T3 (ng/dL)	1.44 *	0.03	1.46	0.11	0.8512
T3 Uptake (%)	28.77	0.38	31.40	1.31	0.0547
T4 (mcg/dL)	11.89 *	0.20	9.99	0.70	**0.0099**
Free T4 (ng/dL)	0.98	0.02	0.80	0.06	**0.0025**
TSH (mU/L)	8.72	0.55	7.69	1.91	0.6072

* *n* = 119. T3, Triiodothyronine; T4, thyroxine; TSH, Thyroid Stimulating Hormone. Bold font reflects statistically different groups.

**Table 8:** Interaction of fetal thyroid hormones with nicotine.

	Nicotine (*n* = 130)			
	Estimate	Std. Error	T-Value	Prob > F
Total T3 * (nmol/L)	1.00	1.157	0.86	0.3893
Free T3 * (ng/dL)	0.78	0.580	1.34	0.1825
T3 Uptake (%)	0.04	0.048	0.90	0.3712
T4 * (mcg/dL)	0.09	0.090	0.95	0.3423
Free T4 (ng/dL)	0.74	1.096	0.68	0.5004
TSH (mU/L)	−0.00	0.033	−0.03	0.9723

* *n* = 129. T3, Triiodothyronine; T4, thyroxine; TSH, Thyroid Stimulating Hormone

**Table 9:** Interaction of fetal thyroid hormone with cotinine.

	Cotinine (*n* = 130)			
	Estimate	Std. Error	T-Value	Prob > F
Total T3 * (nmol/L)	36.48	24.646	1.48	0.1413
Free T3 * (ng/dL)	28.06	12.260	2.29	0.0238
T3 Uptake (%)	−0.36	1.030	−0.35	0.7227
T4 * (mcg/dL)	3.33	1.903	1.75	0.0822
Free T4 (ng/dL)	49.58	23.121	2.14	0.0339
TSH (mU/L)	−0.85	0.710	−1.19	0.2358

* *n* = 129. T3, Triiodothyronine; T4, thyroxine; TSH, Thyroid Stimulating Hormone
